# Comparative quantitative LC–MS/MS analysis of 13 amylase/trypsin inhibitors in ancient and modern *Triticum* species

**DOI:** 10.1038/s41598-020-71413-z

**Published:** 2020-09-03

**Authors:** Sabrina Geisslitz, C. Friedrich H. Longin, Peter Koehler, Katharina Anne Scherf

**Affiliations:** 1 Leibniz-Institute for Food Systems Biology at the Technical University of Munich, Lise-Meitner-Strasse 34, 85354 Freising, Germany; 2 Department of Bioactive and Functional Food Chemistry, Institute of Applied Biosciences, Karlsruhe Institute of Technology (KIT), Adenauerring 20 a, 76131 Karlsruhe, Germany; 3 State Plant Breeding Institute, University of Hohenheim, 70599 Stuttgart, Germany; 4 biotask AG, Schelztorstrasse 54-56, 73728 Esslingen am Neckar, Germany

**Keywords:** Natural variation in plants, Mass spectrometry, Proteins, Proteomics

## Abstract

Amylase/trypsin inhibitors (ATIs) are major wheat allergens and they are also implicated in causing non-celiac gluten sensitivity and worsening other inflammatory conditions. With only few studies on ATI contents in different *Triticum* species available so far, we developed a targeted liquid chromatography-tandem mass spectrometry (LC–MS/MS) method based on stable isotope dilution assays to quantitate the 13 most important ATIs in a well-defined sample set of eight cultivars of common wheat and durum wheat (modern species), as well as spelt, emmer and einkorn (ancient species) grown at three locations in Germany, respectively. Only few ATIs with low contents were detected in einkorn. In contrast, spelt had the highest total ATI contents. Emmer and common wheat had similar total ATI contents, with durum wheat having lower contents than common wheat. Due to the lack of correlation, it was not possible to estimate ATI contents based on crude protein contents. The wheat species had a higher influence on ATI contents than the growing location and the heritability of this trait was high. Despite comparatively low intra-species variability, some cultivars were identified that may be promising candidates for breeding for naturally low ATI contents.

## Introduction

Wheat α-amylase/trypsin-inhibitors (ATIs) are potential triggers of non-celiac wheat/gluten sensitivity (NCWS or NCGS) because of their ability to activate the toll-like receptor 4 TLR4-MD2-CD14 complex on human monocytes, macrophages and dendritic cells causing secretion of proinflammatory chemokines and cytokines^1–3^. This activation of innate immunity is dose-dependent and may result in NCGS symptoms in susceptible individuals as well as worsening of other pre-existing inflammatory reactions^2,4^. The symptoms of NCGS can be both gastrointestinal (e.g., abdominal pain, diarrhea, bloating) and extra-intestinal (e.g., headache, chronic fatigue, depression) and disappear or improve on a gluten-free diet^5–8^. Apart from ATIs, other components have been discussed to cause NCGS, such as gluten peptides and proteins as well as fermentable oligo-, di- and monosaccharides and polyols (FODMAPs)^9–13^. Recently, a study revealed that both a gluten-free diet and a low FODMAP diet reduced the symptoms of NCGS patients, but a gluten-free diet was more effective^14^. FODMAPs might be involved in causing symptoms typical of NCGS^11^, but it was shown that FODMAPs do not trigger the extra-intestinal symptoms that many NCGS patients report^15^. However, gluten peptides and proteins are difficult to exclude as triggers, because commercially available gluten samples and gluten-containing foods contain both ATIs and gluten^16^.

ATIs, known as wheat allergens triggering baker’s asthma^17^, account for about 4% of wheat proteins^18^ and are encoded on at least 19 genes^19^. ATIs are classified into different types either according to their inhibitory activity (amylase and/or trypsin inhibition) or according to their solubility in chloroform/methanol (CM-types). In total, 15 “amylase inhibitors” have evidence at protein level in wheat (UniProtKB: 0.19, 0.28, 0.53, CM1, CM2, CM3, CM16, CM17, CMX1/3, CMX2, wheat amylase subtilisin inhibitor WASI, wheat chymotrypsin inhibitor WCI, xylanase inhibitor, WDAI-3 and allergen C–C). The ATIs 0.19 and CM3 are reported as the two most bioactive ATIs and are the most abundant ATIs (≈ 2.5 mg/g flour, 40% of all ATIs)^1,18–20^. ATIs are resistant to digestive enzymes of insects and mammals due to four to five intramolecular disulfide bonds resulting in a compact three-dimensional structure^19,21–23^.

In general, the content of ATIs can be analyzed by liquid chromatography-tandem mass spectrometry (LC–MS/MS). The stable isotope dilution assay (SIDA) using labeled peptides as internal standards is well established for the absolute quantitation of proteins by LC–MS/MS. The five wheat (sub)species common wheat (*Triticum aestivum* ssp. *aestivum*, hexaploid), spelt (*T. aestivum* ssp. *spelta*, hexaploid), durum wheat (*T. turgidum* ssp. *durum*, tetraploid), emmer (*T. turgidum* ssp. *dicoccum*, tetraploid), and einkorn (*T. monococcum*, diploid) were analyzed for their contents of the predominant ATIs 0.19, 0.28, 0.53, CM2, CM3 and CM16 by SIDA earlier^20^. This showed that einkorn had a very low total ATI content, but spelt and emmer contained higher amounts than common wheat. This was in contrast to the hypothesis that the modern wheat species common wheat and durum wheat have higher ATI contents than the ancient wheat species einkorn, emmer and spelt. Further, this is not consistent to the reduced bioactivity of ATI extracts of ancient wheats (30–70%) compared to common wheat (100%)^1^.

To provide solid comparative data on ATI contents of common wheat, spelt, durum wheat, emmer and einkorn, the first aim of the current study was to expand our quantitation method for six ATIs^20^ to 13 ATIs to cover the total wheat ATI spectrum and to get insights, if previously undetectable ATIs have different contents in the five wheat species. Furthermore, previous work showed that the content of CM3 was influenced both by genotype and environmental conditions^24^. Therefore, the second aim of the current study was to clarify, if the ancient wheats spelt and emmer have a higher ATI content than common wheat independent of the growing location. The third aim was to quantitate the ATIs within each wheat species and relate the intra-species variation to the variation between wheat species. We used a well-defined sample set comprising eight cultivars each of five wheat species all grown next to each other at three different locations, as called for in recent review articles for research on NCGS^25,26^.

## Results

### Identification of ATI marker peptides

The UniProtKB search yielded 15 reviewed proteins, which all originated from *T. aestivum*. No reviewed proteins were present for einkorn, emmer, durum wheat and spelt. Three of the reviewed proteins (P10846, P16852 and P81496) were fragments with a maximal length of 44 amino acids and thus, they were not considered. Further, Q8L5C6 was not included, because it is a xylanase inhibitor. The remaining eleven proteins (Table 1) were appended with the full length protein of CM17 (Q41540) and a wheat trypsin inhibitor (WTI) reported by Altenbach et al*.*^19^, which is similar to A0A1D5UB33. Based on these 13 full-length sequences, suitable marker peptides were chosen for the targeted LC–MS/MS method.

**Table 1:** Overview of the 13 ATIs from wheat quantitated by targeted LC–MS/MS.

UniProtKB accession	UniProtKB name	Abbreviation	Amino acids	Number of theoretical peptides^a^	Number of identified peptides^b^
P01083	Alpha-Amylase Inhibitor 0.28	0.28	153	9	7
P01085	Alpha-Amylase Inhibitor 0.19	0.19	124	7	7
P01084	Alpha-Amylase Inhibitor 0.53	0.53	124	7	6
P16850	Alpha-Amylase/Trypsin Inhibitor CM1	CM1	145	5	3
P16851	Alpha-Amylase/Trypsin Inhibitor CM2	CM2	145	4	2
P17314	Alpha-Amylase/Trypsin Inhibitor CM3	CM3	168	9	5
P16159	Alpha-Amylase/Trypsin Inhibitor CM16	CM16	143	5	4
Q41540	CM17 Protein	CM17	143	4	4
P16347	Endogenous Alpha-Amylase/Subtilisin Inhibitor	WASI	180	8	6
Q43723	Trypsin/alpha-Amylase Inhibitor CMX1/CMX3	CMX1/3	121	5	1
Q43691	Trypsin/alpha-Amylase Inhibitor CMX2	CMX2	121	5	1
P83207	Chymotrypsin Inhibitor	WCI	119	3	3
–^c^	Wheat Trypsin Inhibitor	WTI	137	7	3

^a^Derived from in silico tryptic digestion with a minimal number of eight and a maximal number of 26 amino acids.

^b^Identified by at least five transitions at the same retention time. For amino acid sequences of all identified peptides see Supplementary Table S1.

^c^Sequence according to Altenbach et al*.*^19^; similar to UniProtKB number A0A1D5UB33 (AAI domain-containing wheat protein), 70% identity.

The in silico digestion of the 13 ATIs resulted in three (WCI) to nine (0.28) tryptic peptides per ATI and 78 in total (Table 1). Of these, LC–MS/MS screening of the tryptic ATI extract from the wheat flour mix provided 46 peptides with at least five transitions at the same retention time (see Supplementary Table S1 and data on Panorama Public). Only one peptide was detectable for CMX1/3 and CMX2, but all seven peptides for 0.19 and all four peptides for CM17. For 0.28, 0.53, CM1, CM2, CM3, CM16, WASI, WCI and WTI, 43–86% of the theoretical peptides were identified. Due to the high similarity of certain ATIs, five peptides were present both in 0.19 and 0.53, one peptide both in CM16 and CM17 and the one peptide of CMX1/3 and CMX2 in both ATIs (named CMX1/2/3 in the following).

We aimed to select two peptides per protein, because of possibly unknown and variable posttranslational modifications. This was not possible for 0.53 (only one unique peptide to differentiate 0.53 from 0.19), CM1 and CM17 (low intensities) and CMX1/2/3 (no other peptide detected). For the other ATIs, two peptides were selected resulting in 20 marker peptides (P1-P20) to quantitate 13 wheat ATIs (Table 2).

**Table 2:** Targeted LC–MS/MS parameters for the quantitation of 13 wheat ATIs.

Peptide/internal standard	ATI	Amino acid sequence^a^	Precursor *m/z*		Product ions	Collision energy (eV)	Retention time (min)
			P	IS			
P1/IS1	0.28	LQ**C**VGSQV*PEA*VLR	778.4	784.4	b3/y6/y10/y11/y12	24/23/26/26/25	14.6
P2/IS2	0.28	LTAASVPEVC*K	587.8	591.8	y2/y5/y7/y8/y9	26/18/17/17/17	11.1
P3/IS3	0.19 + 0.53	LQ**C**NGSQV*PEA*VLR	785.9	791.9	y4/y6/y7/y8/y10	29/23/25/22/27	13.4
P4/IS4	0.19 + 0.53	LTAASITAVC*R	581.8	586.8	y4/y5/y7/y8/y9	16/15/16/16/16	12.6
P5/IS5	0.53	EHGVSEGQAGTGAFPSC*R	616.3^+^	619.6^+^	b2/b3/y4/y5/y7	29/30/21/21/21	10.0
P6/IS6	CM1	SDPNSSVL*K	473.7	477.8	b2/y4/y5/y6/y7	12/17/15/17/12	7.7
P7/IS7	CM2	EYVAQQT**C**GVGIVGSPVSTE*P*GNT*PR	901.8^+^	906.8^+^	b3/y6/y9/y11/y13	36/35/32/34/33	14.2
P8/IS8	CM2	TSDPNSGVL*K	509.3	513.3	b3/y5/y6/y7/y8	13/17/14/14/13	7.6
P9/IS9	CM3	YFIALPVPSQPVDP*R	850.0	855.0	y2/b3/b4/y8/y10	33/27/23/23/23	19.4
P10/IS10	CM3	SGNVGESGLIDL*PGC*PR	864.4	870.4	y6/y7/y10/y11/y13	29/24/25/25/25	15.9
P11/IS11	CM16	DYVEQQAC*R	584.8	589.7	b2/y5/y6/y7	19/17/19/21/17	8.3
P12/IS12	CM16	QQ**CC**GE*LANI*PQQ**C**R	931.4	937.9	y5/y6/y7/y8/y9	27/28/26/26/26	11.8
P12c/IS12c	CM16	**Q**Q**CC**GE*LANI*PQQ**C**R	922.9	929.4	y5/y6/y7/y8/y9	27/26/27/27/29	13.3
P13/IS13	CM17	NYVEEQAC*R	584.8	589.8	b3/y5/y6/y7	19/17/19/21	8.6
P14/IS14	WASI	HVITGPV*R	439.8	444.8	b1/b2/y4/y5/y6	20/15/15/14/13	7.4
P15/IS15	WASI	YSGAEVHEY*K	591.8	595.8	y4/y5/y6/y8/b8	19/18/19/17/18	8.0
P16/IS16	CMX1/2/3	EFIAGIVG*R	481.3	486.3	y3/y4/y5/y6/y7	14/15/15/15/15	15.4
P17/IS17	WCI	ELAAISSNC*R	560.8	565.8	b3/y5/y6/y7/y8	17/18/18/18/18	9.8
P18/IS18	WCI	AFPPSQSQGGGPPQPPLAP*R	993.5	998.5	y3/y6/y8/y9/y12	38/34/31/33/35	15.5
P19/IS19	WTI	ELEAVSEEC*R	611.3	616.3	y3/y5/y6/y7/y8	19/19/19/19/22	9.6
P20/IS20	WTI	LEGVPEG**C**T*R	559.3	564.3	b2/b4/y5/y6/y8	18/19/25/23/16	9.4

^a^**C**, S-carboxamidomethylcysteine; **Q**, pyroglutamic acid; *P, proline (^13^C_5_, ^15^N); *V, valine (^13^C_5_, ^15^N); *K, lysine (^13^C_6_, ^15^N_2_); *R, arginine (^13^C_6_, ^15^N_4_) *G, glycine (^13^C_2_, ^15^N); *L, leucine (^13^C_6_, ^15^N).

^+^Precursors were 3^+^, all other ones 2^+^.

### Development of SIDA

Ideally five selected reaction monitoring (SRM) transitions per marker peptide were taken including the four most abundant transitions and one transition with a product ion mass-to-charge ratio (*m/z)* higher than the *m/z* of the precursor ion to improve the selectivity. P11 and P13 had only four transitions in the final method due to matrix interferences. The final SRM transitions and their optimized collision energies (CE) are shown in Table 2. For each of the 20 marker peptides, the respective labeled internal standards (IS1-20) were selected and the same SRM transitions and corresponding CE were included in the final method. Applying the final optimized and timed LC–MS/MS method resulted in 18 to 34 scan events per peak.

### Method validation

Precision, limits of detection (LOD), limits of quantitation (LOQ) and recovery of the SIDA were evaluated (Table 3). The values for repeatability (1.0–7.6%) were very good for all peptides. The analysis of the peptides also showed good intermediate precision (2.3–9.6%) in most cases. The comparatively poor intermediate precision (≤ 15.6%) for P15, P17, P18 and P19 was due to low concentrations in wheat flour, that of P7 most likely due to its peptide length (26 amino acids) and that of P12c due to the formation of *N*-terminal pyroglutamic acid.

**Table 3:** Validation parameters of the targeted LC–MS/MS method for the quantitation of 13 wheat ATIs.

Peptide	ATI	Amino acid sequence^a^	Precision (%)^b^		Sensitivity peptide (µg/g)		Sensitivity protein (µg/g)		Recovery^e^
			Repeatability	Intermediate	LOD^c^	LOQ^d^	LOD^c^	LOQ^d^	
P1	0.28	LQ**C**VGSQVPEAVLR	2.9	3.2	0.43	1.43	3.75	12.50	110.0
P2	0.28	LTAASVPEV**C**K	1.0	2.3	1.73	5.76	20.26	67.55	105.3
P3	0.19 + 0.53	LQ**C**NGSQVPEAVLR	2.0	4.5	0.15	0.51	1.35	4.51	103.4
P4	0.19 + 0.53	LTAASITAV**C**R	3.3	8.4	0.62	2.08	7.50	25.10	114.7
P5	0.53	EHGVSEGQAGTGAFPS**C**R	4.8	5.5	0.76	2.55	5.63	18.76	104.7
P6	CM1	SDPNSSVLK	3.1	4.3	0.20	0.67	2.80	9.33	101.0
P7	CM2	EYVAQQT**C**GVGIVGSPVSTEPGNTPR	5.5	14.5	2.56	8.53	12.62	42.07	93.1
P8	CM2	TSDPNSGVLK	1.8	3.6	0.15	0.48	1.86	6.21	103.3
P9	CM3	YFIALPVPSQPVDPR	2.4	2.8	0.05	0.16	0.44	1.47	110.1
P10	CM3	SGNVGESGLIDLPG**C**PR	3.4	7.0	0.19	0.63	1.80	6.00	112.8
P11	CM16	DYVEQQA**C**R	3.1	4.0	0.63	2.10	7.65	25.50	102.2
P12	CM16	QQ**CC**GELANIPQQ**C**R	3.6	4.4	3.92	13.07	31.13	103.77	103.8
P12c	CM16	**Q**Q**CC**GELANIPQQ**C**R	5.4	13.5	1.21	4.03	9.74	32.47	107.5
P13	CM17	NYVEEQA**C**R	3.0	5.1	0.20	0.67	2.42	8.07	102.6
P14	WASI	HVITGPVR	5.1	7.2	0.10	0.33	2.23	7.43	150.4
P15	WASI	YSGAEVHEYK	7.5	14.7	1.09	3.63	18.11	60.37	130.2
P16	CMX1/2/3	EFIAGIVGR	3.2	8.0	0.19	0.63	2.26	7.53	124.4
P17	WCI	ELAAISSN**C**R	7.6	12.7	0.88	2.93	9.43	31.43	113.2
P18	WCI	AFPPSQSQGGGPPQPPLAPR	3.9	12.8	0.61	2.03	3.53	11.77	121.3
P19	WTI	ELEAVSEE**C**R	5.2	15.6	0.14	0.46	1.75	5.84	108.7
P20	WTI	LEGVPEG**C**TR	6.8	9.6	0.97	3.23	13.40	44.67	153.5

^a^**C**, S-carboxamidomethylcysteine; **Q**, pyroglutamic acid.

^b^Precision in common wheat flour, determined with a wheat flour mix of 15 cultivars: Repeatability: Six replicates in one day; intermediate: Six replicates in three days.

^c^LOD, limit of detection, using gluten-free wheat starch as matrix.

^d^LOQ, limit of quantitation, using gluten-free wheat starch as matrix.

^e^Recovery in wheat flour diluted with gluten-free wheat starch (1 + 4).

Gluten-free wheat starch was used as matrix to determine LOD and LOQ, for which the absence of marker peptides and interferences had been confirmed by prior LC–MS/MS analysis. The marker peptides were detected with high sensitivity, resulting in an LOD between 0.1 and 3.9 µg/g and an LOQ between 0.2 and 13.1 µg/g. These limits agreed well with those reported for gluten peptides^27^ and for ATI peptides in our previous study^20^. The calculated LOD and LOQ were confirmed by spiking the exact concentrations of LOD and LOQ to gluten-free wheat starch. The concentrations at LOD still fulfilled the identification criteria, because the peak area ratios of the SRM transitions were constant. The LOD and LOQ of the proteins were calculated by multiplying the peptide contents with the protein-specific factor considering the molecular weights of the peptide and the protein. Except for P12 (LOD, 31.1 µg/g, LOQ, 103.8 µg/g), very low LOD (1.4–20.3 µg/g) and LOQ (4.5–67.5 µg/g) were determined.

The recovery was analyzed by comparing the ATI contents of undiluted wheat flour with those of wheat flour diluted with gluten-free wheat starch (1 + 4). The advantage of this procedure compared to classical spike experiments is that the ATIs are present as proteins in flour, in contrast to spiking experiments that use the peptides and do not represent the real protein recovery. The recovery was determined as comparison between the analyzed ATI contents in diluted flour and the expected ATI contents calculated from the undiluted flour. In general, the recovery was between 93.1% and 113.2% for the majority of peptides. This confirmed that the ATIs were extracted in both undiluted and diluted flour in similar amounts with our extraction procedure. P14, P15, P16, P18 and P20 showed higher recoveries (≤ 153.5%). The reason for this was that the content of these peptides was generally low in flour and that the dilution of 1 + 4 was close to the LOQ.

### Detection of ATI marker peptides in the five wheat species

In general, the einkorn cultivars contained only few ATIs (Supplementary Table S2). P4 (0.19), P9 and P10 (CM3) and P19 (WTI) were detectable in three, six and eleven out of 24 einkorn samples, respectively. P14 (WASI) and P16 (CMX1/2/3) were detectable in all samples, but not the second peptide of WASI (P15). No other marker peptides were detected in the einkorn samples. In contrast, almost all peptides were detectable in emmer and durum wheat except the peptides of CM17 (P13) and WCI (P17 and P18). Further, P6 (CM1) was present in only two out of eight emmer cultivars at one location and in the eight durum wheat samples of one location (Seligenstadt). P1 and P2 (0.28) were only detectable in two durum wheat cultivars of all locations. All peptides were detected in the hexaploid wheat species common wheat and spelt.

### Quantitation of 13 ATIs

The ATI content of flours of eight cultivars of common wheat, spelt, durum wheat, emmer and einkorn grown in the same year (2013) at three different locations in Germany is shown in detail in Supplementary Table S2. Only one peptide was available to quantitate the ATIs 0.53, CM1, CM17 and CMX1/2/3. In contrast, two peptides were available to calculate the protein content for the ATIs 0.19, 0.28, CM2, CM3, CM16, WASI, WCI and WTI. Differences between the contents of ATIs were observed depending on the peptide used for calculation (e.g., P3 or P4 for 0.19). For the hexaploid wheat species common wheat and spelt, the content of all ATIs calculated with the two respective peptides was highly correlated (r ≥ 0.712) (see Supplementary Table S3). However, the content of 0.19 calculated using P3 and P4 was not correlated for the tetraploid wheat species durum wheat (r = 0.110) and emmer (r = 0.526) and that of CM16 based on P11 and P12 was not correlated for emmer (r = 0.059). These results indicated that 0.19 and CM16 might be modified by posttranslational modifications, especially in tetraploid wheat species.

In order to compare the total ATI content between wheat species, one peptide per ATI was selected. In most cases it was irrelevant which peptide was used due to the high correlation of the content of both peptides for one ATI. Thus, one peptide was selected as “quantifier” and the second one as “qualifier” to ensure correct identification of the respective ATI. Of the two, the peptide with fewer amino acids was chosen as quantifier, respectively. However, the selection of a peptide, which is suitable for quantitation (“quantotypic”) is challenging and should fulfill several criteria (see “Discussion”)^28,29^. Thus, P2 was selected for 0.28, P4 for 0.19, P8 for CM2, P9 for CM3, P11 for CM16, P14 for WASI and P17 for WCI. Both peptides of WTI had ten amino acids and P19 was selected due to a lower LOD than P20.

**Figure 1:**
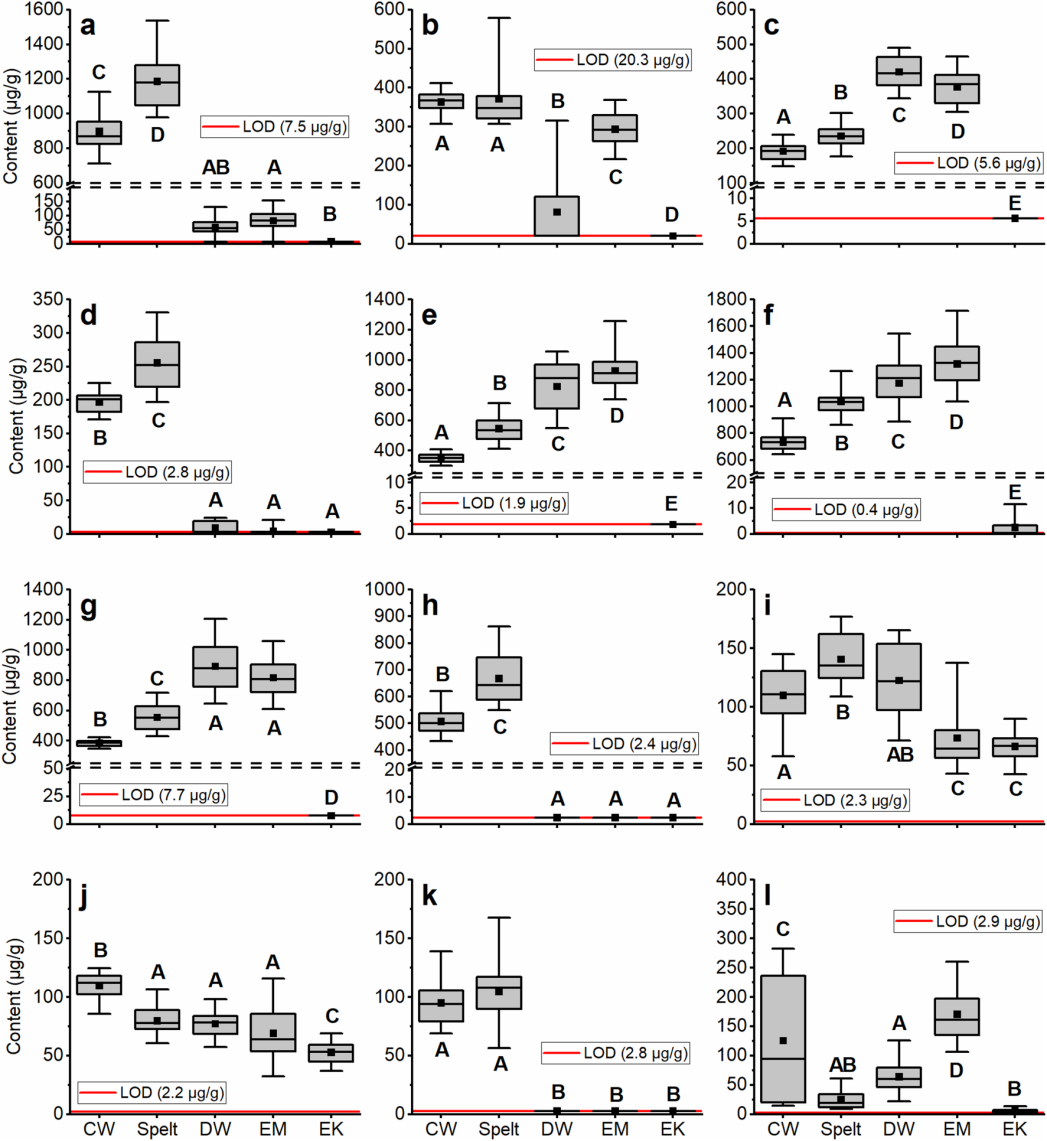
Contents of ATIs (µg/g) based on flour weight. **(a)** 0.19; **(b)** 0.28; **(c)** 0.53; **(d)** CM1; **(e)** CM2; **(f)** CM3; **(g)** CM16; **(h)** CM17; **(i)** CMX1/2/3; **(j)** WASI; **(k)** WCI; **(l)** WTI. Box plots represent the median (line in the box), mean (point in the box) and the 25% and 75% percentiles. The whiskers show the minimum and maximum values. Boxes with different capital letters indicate significant differences between the wheat species (one-way ANOVA with Tukey’s test, p < 0.05). Each box summarizes the content of 24 samples (eight cultivars grown at three locations, respectively). *CW* common wheat, *DW* durum wheat, *EM* emmer, *EK* einkorn, *LOD* limit of detection.

No matter which peptide was used for 0.19, emmer and durum wheat had significantly lower 0.19 contents than common wheat and spelt (Fig. 1a). Nevertheless, using P4 the contents of 0.19 were very low in emmer and durum wheat (< 150 µg/g) or very close to the LOD. Using P3, the contents were about 400 µg/g higher for all wheat species except einkorn. In addition to modifications of the peptides, this observed difference might be due to the high content of P3, which was at the upper end of the calibration line.

The content of 0.28 was higher in common wheat and spelt than in emmer (Fig. 1b). Both peptides of 0.28 were detectable in only two durum wheat cultivars (Lunadur and Wintergold), but with similar levels as in emmer. Incidentally, the cultivar Wintergold is the most cultivated durum wheat cultivar in Austria and Germany. Apparently some durum wheat cultivars do not express 0.28 and this is in accordance with our previous study^20^. In contrast to 0.19 and 0.28, the content of 0.53 was significantly higher in durum wheat and emmer than in common wheat and spelt (Fig. 1c).

As already stated, CM1 and CM17 were either not detectable in emmer and durum wheat or the contents were very low, whereas common wheat and spelt had high concentrations (Fig. 1d and h). For the other CM-types CM2, CM3 and CM16 (Fig. 1e, f and g), a similar trend was observed with a lower content in hexaploid wheat species (common wheat < spelt) than in tetraploid wheat species (durum wheat ≤ emmer). The comparison of the CM16 content calculated with two different peptides showed that some emmer cultivars had a very low content of P12, but the mean and median values over all emmer cultivars were comparable to those of P11.

In general, the ATIs WASI, CMX1/2/3, WCI and WTI were less abundant than the major ATIs 0.19, 0.28, 0.53, CM1, CM2, CM3, CM16 and CM17. CMX1/2/3 and WASI were the only two ATIs, which had a similar content in all five wheat species including einkorn (Fig. 1i, j). Common wheat had the highest WASI content and emmer and einkorn the lowest. Spelt and durum wheat had a higher CMX1/2/3 content than emmer and einkorn. WCI was only detectable in common wheat and spelt with no differences between both wheat species (Fig. 1k). The variation of the WTI content in common wheat was very high among cultivars and ranged from < 200 µg/g to < 25 µg/g (Fig. 1l). The WTI content was higher in emmer than in spelt and durum wheat, but einkorn contained very low amounts.

### Total ATI content

**Figure 2:**
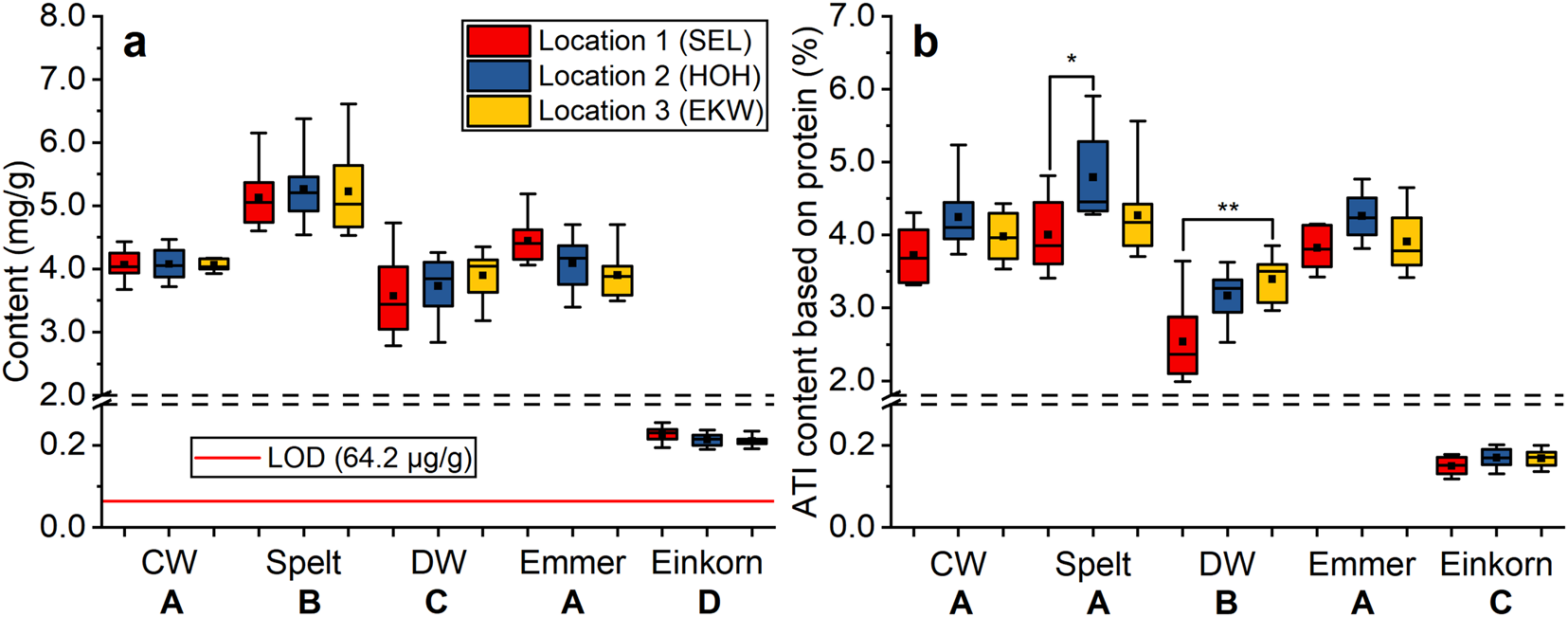
Total ATI contents (mg/g) **(a)** and percentage of the total ATI content relative to total protein content **(b)** of eight cultivars per box grown at the three locations Seligenstadt (SEL), Hohenheim (HOH) and Eckartsweiher (EKW). Boxes, lines and whiskers are presented as in Fig. 1. Boxes marked with asterisks are significantly different (one-way ANOVA with Tukey’s test, * p < 0.05, ** p < 0.01) and different capital letters below the x-axis indicate significant differences between the wheat species.

As previously described^20^, the ancient wheat species spelt had a significantly higher total ATI content than the modern wheat species common wheat and durum wheat (Fig. 2a). This was true independent of the growing location. The total ATI content of einkorn was less than 5% compared to the other wheat species. In contrast to our previous work, the ancient wheat species emmer had a similar total ATI content as modern common wheat, because the ATI content tended to be lower at Hohenheim and Eckartsweiher compared to Seligenstadt.

No differences of the total ATI content based on the protein content between common wheat, spelt and emmer were observed, because spelt had higher protein and higher total ATI content than common wheat and emmer (Fig. 2b). Based on the protein content, durum wheat had a lower ATI content than common wheat, spelt and emmer. However, the protein content (Supplementary Table S2) was not correlated with the ATI content over all five wheat species (r = − 0.049), for four wheat species except einkorn (r = 0.193) or for each wheat species separately (common wheat r = 0.271; spelt r = 0.290; durum wheat r = − 0.186; emmer r = 0.586). Thus, it is not possible to predict the ATI content from the total protein content and this shows the high necessity of our method.

### ATI distribution

**Figure 3:**
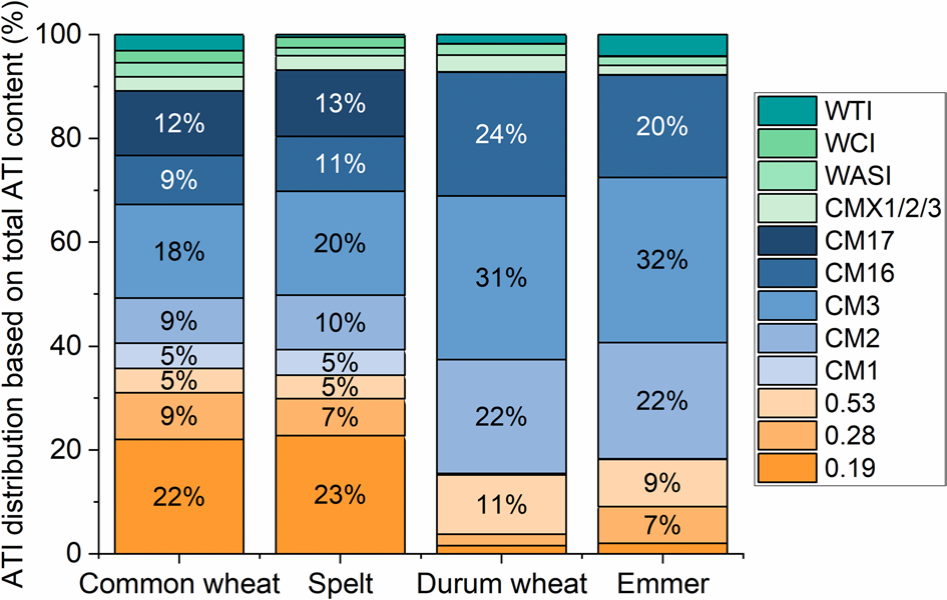
Average distribution of the 13 ATIs based on total ATI contents for common wheat, spelt, durum wheat and emmer (24 samples per wheat species). Percentages below 4.5% were not explicitly marked for better readability.

**Figure 4:**
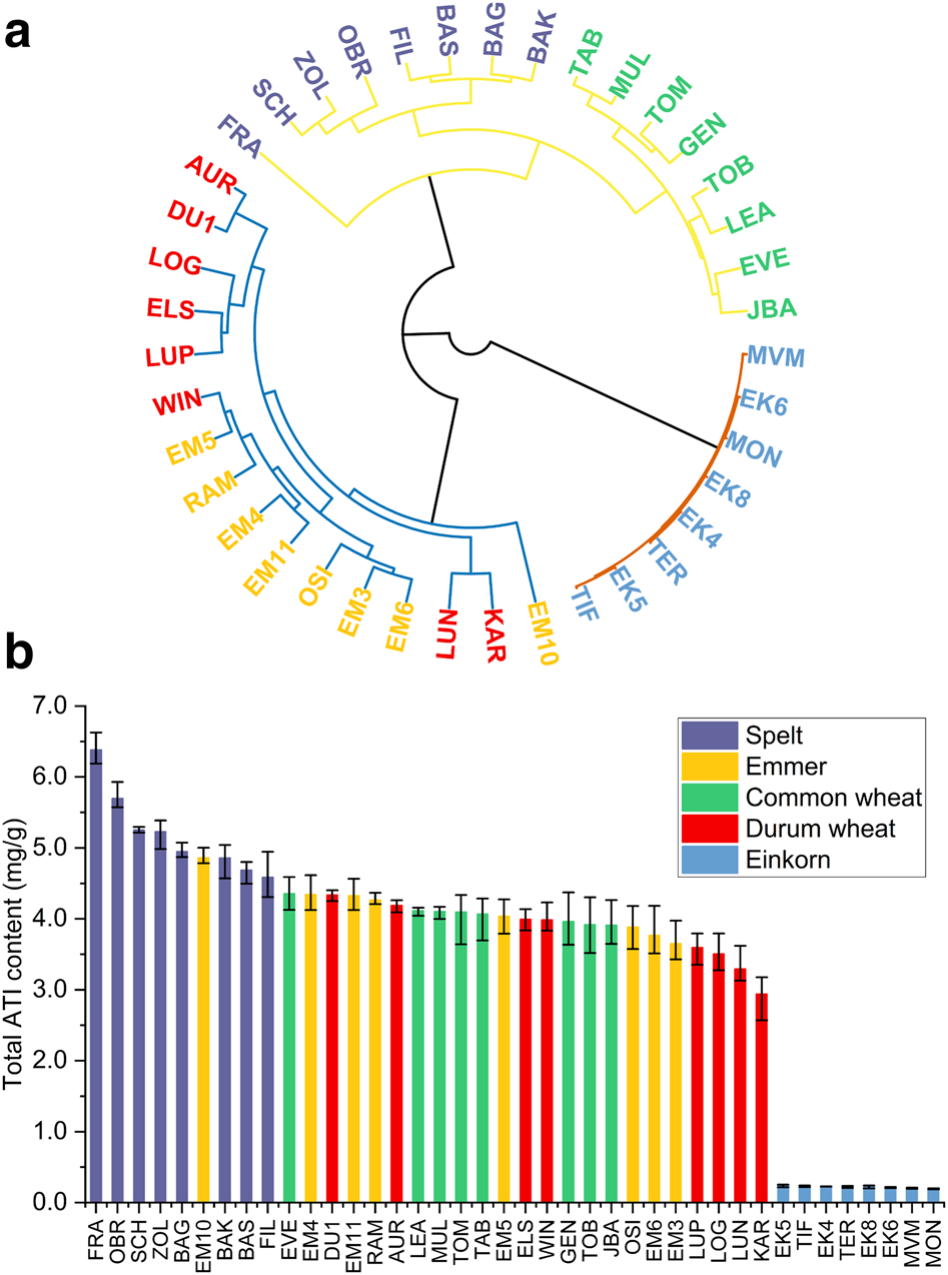
**(a)** Hierarchical cluster analysis including the mean absolute ATI contents of the three locations. Three clusters were identified according to the ploidy level of the *Triticum* species: hexaploid, yellow; tetraploid, blue and diploid, red connecting lines. The single cultivars of the different wheat species are colored as in **(b)** and the abbreviations are shown in Supplementary Table S2. **(b)** Total ATI content (mg/g) per cultivar grown at the three locations, presented as mean value and error bars show the respective minimal and maximal content.

The ATI distribution of the wheat species differed according to their ploidy levels. The ratio between the ATIs 0.19, 0.28 and 0.53 and the CM-types was more balanced in the hexaploid wheat species than in the tetraploid wheat species (Fig. 3). The ATIs 0.19, 0.28 and 0.53 accounted for approximately 36% (32–40% for the eight cultivars of three locations) of all ATIs in common wheat, for 34% (33–37%) in spelt, for 15% (11–22%) in durum wheat and for 18% (16–21%) in emmer. In contrast, the CM-types corresponded to 53% (50–58%) in common wheat, to 59% (55–62%) in spelt, to 78% (72–83%) in durum wheat and to 74% (70–77%) in emmer. The significant difference in the ATI distribution was confirmed by hierarchical cluster analysis (Fig. 4a). Three main clusters were observable according to the ploidy levels (hexaploid, tetraploid and diploid). The cluster of the hexaploid wheat species was further subdivided into three clusters: the spelt cultivar Franckenkorn, the other seven spelt cultivars and all common wheat cultivars. This showed lower variability within wheat species than between wheat species. The special position of Franckenkorn was because of its very high ATI content. Except for one emmer cultivar, all emmer cultivars were clustered in between the cluster of tetraploid wheat species. The durum wheat cultivars were not clearly clustered showing higher variability within tetraploid wheat species than within hexaploid wheat species.

### Influence of the growing location and wheat species

For the majority of the ATIs, there were no significant differences between the three locations for each wheat species according to one-way analysis of variance (ANOVA). Significant differences between the three locations (p < 0.05) were present for 0.53 (emmer and durum wheat), CM3 (emmer) and WCI (common wheat and spelt). Because there were so few significant differences between locations for the ATIs, the data are not displayed for each location, but are grouped into one box plot in Fig. 1. In addition, for the total ATI content (Fig. 2a), no differences between different locations were observed. However, the ATI content based on the protein content differed significantly between Seligenstadt and Hohenheim for spelt and between Seligenstadt and Eckartsweiher for durum wheat (Fig. 2b).

Two-way ANOVA with the factors location and wheat species confirmed the results of one-way ANOVA. The factor wheat species had a higher influence on the absolute content than the factor location for the majority of the ATIs (see Supplementary Table S4). WCI was an exception, because the influence of the location was higher than that of the wheat species. Further, a significant influence of the location and an interaction of both factors was observed only for 0.53.

Considering the ATI content relative to the total protein content, the influence of the location on 0.28, 0.19, 0.53, CM2, CM3, CM16, WASI, CMX1/2/3, WTI and total ATI content was very low in comparison to that of the wheat species. The influence of the factors location and wheat species was almost equal for CM1, CM17 and WCI, because these ATIs were only present in hexaploid wheat species and values below the LOD were not considered for two-way ANOVA. Similar results were obtained for the ATI distribution, irrespective of whether the ATI content was calculated relative to total ATI content or total protein content. This showed that the ploidy level had a higher influence on the distribution of ATIs than the location.

### Heritability

The heritability (h^2^) describes the amount of genetic variance relative to the total variance observed in the field, which is the sum of genetic and environmental variance for the measured traits (here: content of ATIs and total ATI). In general, the heritability is between 0–1 and the higher the heritability, the higher the genetic effect. With a high heritability, specific cultivars can be selected to provide special properties to consumers, e.g., reduced ATI contents.

We identified a very high heritability for all ATIs in spelt (h^2 ^≥ 0.88) and all detectable ATIs in emmer (h^2 ^≥ 0.86 and h^2 ^= 0.62 for 0.53) (Table 4). With the exception of 0.19, 0.28 and 0.53, the heritability was also very high for durum wheat (h^2 ^≥ 0.79). In einkorn only CMX1/2/3 and WASI were present in all cultivars showing a very high heritability (h^2 ^≥ 0.87). In contrast, the heritability in common wheat was not as clear as in the other wheat species. Only CM2, CM3, CMX1/2/3, WCI and WTI had very high heritability (h^2 ^≥ 0.83) and 0.28, 0.19, 0.53, CM1 had high heritability (h^2 ^≥ 0.58). According to the heritability, the ancient wheat species einkorn, emmer and spelt represent promising candidates in further breeding for reduced ATI contents.

**Table 4:** Heritability (h^2^) of the contents of ATIs and total ATI based on eight cultivars of each wheat species grown at three locations.

ATI	Common wheat	Spelt	Durum wheat	Emmer	Einkorn
0.28	0.73	0.99	0.14	0.98	–
0.19	0.58	0.95	0.68	0.89	–
0.53	0.67	0.93	0.00	0.62	–
CM1	0.72	0.97	–	–	–
CM2	0.84	0.97	0.93	0.97	–
CM3	0.83	0.92	0.81	0.94	0.28
CM16	0.00	0.98	0.93	0.86	–
CM17	0.30	0.99	–	–	–
WASI	0.79	0.88	0.79	0.90	0.87
CMX1/2/3	0.98	0.93	0.94	0.99	0.92
WCI	0.91	0.95	–	–	–
WTI	0.83	0.88	0.92	0.96	0.65
Total ATI	0.47	0.67	0.90	0.97	0.87

Missing values (–) were not calculated because the contents were lower than the limit of detection.

### Cultivars with low ATI contents

The major ATIs 0.19, 0.28, 0.53, CM1, CM2, CM3, CM16 and CM17 were not detectable in any einkorn cultivar at the location Eckartsweiher. Further, these ATIs were not detectable in the two einkorn cultivars M-04033/03 (EK6) and M-07006/01 (EK8) at all three locations making them promising candidates for further breeding (Supplementary Table S2).

The common wheat cultivar Genius and the spelt cultivar Filderstolz contained low amounts of total ATI (Fig. 4b) and of the two most bioactive ATIs 0.19 and CM3 compared to the other cultivars at all three locations (5–10% less compared to respective mean value), although the total protein content was comparable to the other cultivars. The durum wheat cultivars Karur and Lunadur and the emmer cultivar Teutonia had at least 10% lower CM3 and total ATI levels compared to the respective mean value. The content of 0.19 was very low for all durum wheat and emmer cultivars.

## Discussion

We developed a new LC–MS/MS method based on SIDA for the absolute quantitation of the 13 most important ATIs in wheat. As alternative source for our customized database, the amino acid sequences of 19 ATIs in the common wheat cultivar Butte 86, which were analyzed by untargeted LC–MS/MS after gel electrophoresis, are available in Altenbach et al*.* (2011)^19^. The comparison of the amino acid sequences of our database containing 13 ATIs with those of Butte 86 showed only small differences for the majority of ATIs (see Supplementary Table S5). The reason for the difference in the number of ATIs was that two isoforms were identified for 0.19, 0.28, 0.53, CM3, WASI and CMX1/3 in Butte 86, respectively. The greatest differences were observed for the amino acid sequences of both isoforms of CMX1/3 and of CMX2. However, our marker peptides were present in all amino acid sequences of 18 ATIs with the exception of one isoform of 0.53. Thus, the content of our study represents the content of the ATIs of Butte 86. Apart from this, the amino acid sequence of the WTI reported in Altenbach et al*.* (2011)^19^ was included in the final method. The amino acid sequence of this WTI was similar to that of the UniProtKB accession A0A1D5UB33 (57 additional amino acids at the *N*-terminus and an additional T at position 173 of A0A1D5UB33) listed as “AAI domain-containing protein” since July 2019. Probably, this protein would have not been considered in untargeted LC–MS/MS searches due to its special name without the keywords “amylase” and “inhibitor”.

The selection of marker peptides, which are representative for a protein, is afflicted with certain restrictions^28^. Besides the peptide length (8–26 amino acids), the marker peptides should be unique, ideally contain no cysteine (possibly incomplete alkylation), methionine and tryptophan (partial oxidation) and have good MS detectability. Most of the identified peptides were not only present in the genus *Triticum*, but also in other genera of the *Triticeae* tribe (e.g., *Aegilops*) or other cereals (rye and barley) due to extensive amino acid sequence homologies. This is why our selection criterion for uniqueness was based on the differentiation to gluten proteins and proteins of other organisms (e.g., plants other than *Poaceae*, animals, yeast or cleavage enzymes), and thus, the peptides were unique for ATIs present in the *Poaceae* family. Because ATIs are rich in cysteine, the majority of the identified peptides contained one or more cysteine residues. However, a good MS detectability characterized by high intensities and few matrix interferences was more important than the absence of cysteine, because reduction and alkylation were performed, which were assumed to be quantitative.

To differentiate 0.19 and 0.53, the unique peptide of 0.53 (P5) was selected, but no unique peptide of 0.19, although two peptides were detected during method development (Supplementary Table S1). The reasons were that one of these peptides showed very low intensities and the other one was influenced by matrix interferences. Thus, we decided to include two abundant and well-detectable peptides, which represented the sum of 0.19 and 0.53 and a third peptide of 0.53 to differentiate both proteins. In a new method or in relative quantitation methods (see below) more peptides might be considered to increase method performance. Due to the high similarity of the amino acid sequences, CMX1/3 and CMX2 could not be distinguished with our method. CMX1/3 and CMX2 differ only in one amino acid (position 97 G → D) and the peptide carrying this substitution was not detectable by targeted LC–MS/MS.

For the ATIs 0.28, 0.19, CM2, CM3, CM16, WCI and WTI, two marker peptides were available to calculate the absolute content of the corresponding ATI, which yielded different results. This issue is a general problem in absolute quantitation of proteins using labeled peptide standards. The selection of peptides, which represent the absolute content of proteins (“quantotypic” peptides), is afflicted with further special requirements^29^. The peptide content is influenced by amino acid modifications, by changes in only a part of the proteins or by incomplete digestion, which occurs, e.g., due to incomplete denaturation or specific amino acid combinations^28,30^. Further, peptides have a limited stability in solution. It is recommended, e.g., to minimize freeze/thaw cycles, to store the peptides either as solutions with high concentrations at − 80 °C or lyophilized at − 20 °C and use silanized glass vessels^30^. These recommendations were carefully followed during this study. The time point at which the IS are introduced also affects the peptide content^31^. We used the “pre-digest” method, because the IS should be added as early as possible and should be reduced and alkylated simultaneously with the native peptides to account for losses during sample preparation. In comparison to the “concurrent” method, where the IS are added together with trypsin, the pre-digest method can lead to overestimation, because the IS will decay more rapidly during digestion than the native peptide. However, the “post-digest” method when the IS are added prior to analysis leads to high rates of underestimation, because the native peptide will be subjected to degradation but not the IS. An option to avoid these effects would be to use full-length protein standards for absolute quantitation (PSAQ)^32^. However, this method is very costly, time consuming and also does not correct for modifications of the natural protein^28,33^.

In this study, the contents of two peptides used to quantitate the ATIs 0.28, 0.19, CM2, CM3, CM16, WCI and WTI were calculated. With the exception of 0.19 in durum wheat and emmer and CM16 in emmer, the contents using either the first or second peptide were highly correlated (Supplementary Table S3). However, using the respective other peptide of 0.19 and CM16, resulted still in a similar total ATI content and the ratio between both contents was constant at 1.1–1.2. The main statements that (i) spelt has higher total ATI contents than common wheat and (ii) emmer and common wheat have higher total ATI contents than durum wheat did not change irrespective of the peptide used for calculation.

Relative methods, e.g., label-free quantitation (LFQ), are alternatives to absolute quantitation combined with SIDA. Possible options are data-dependent acquisition (DDA) followed by data evaluation using algorithms such as “intensity based absolute quantitation” (iBAQ)^28,34^ or SRM monitoring without labelled peptides. However, SIDA is more precise, accurate and sensitive than relative methods showing the advantage of our new method compared to relative quantitation^28^.

Recently, 18 ATIs, including isoforms of 0.19, 0.28, CM1, CM2, CM3, CM16 and CMX1/3, were monitored by SRM in several common wheat samples^35^. With this approach, it was possible to relatively compare different samples, but no absolute content can be calculated. A variability of 60–125% based on the median was observed in the total ATI content of the 15 common wheat cultivars studied. In our study, the common wheat cultivars showed a lower variability of only 90–110% even considering all three locations. As protein content and composition are influenced by growing conditions, one reason might be that the climatic conditions in Germany varied to a lower extent than in the Australian study. One interesting aspect was that one Australian common wheat cultivar seemed to be a promising candidate for breeding of “low-ATI-wheat”. However, all common wheat samples in our study had comparable ATI contents. Even if high heritability was observed, no common wheat cultivar analyzed seems to have the high potential of the Australian one. However, some durum wheat (Karur and Lunadur) and emmer (Teutonia) cultivars might be promising candidates for further breeding, especially for pasta making, due to a low content of total ATI, 0.19 and 0.28.

The ATI encoding genes were already identified in common wheat^36^, but not in tetraploid and diploid wheat species, so that relations between the encoding genes and the expressed ATIs would be premature. Only CMX1/2/3 and WASI were detectable in diploid einkorn (genome A^m^A^m^), confirming that the other ATI genes might be silenced in einkorn lines^37^. Only one peptide (P15) was detectable for WASI in all einkorn cultivars, but not P14. This was probably due to the much higher LOD of P14 compared to P15, but modifications could also be a reason for this.

The heritability of the ATI content was, in general, slightly lower compared to that of agronomic performance^38^ and gluten protein composition^39^. However, the majority of the ATI contents had high heritability. This confirmed again the high quality of the field trials and the robustness of our results, even though the sample set was cultivated in the same year.

To sum up, the LC–MS/MS method based on SIDA performs well in terms of precision, detection limits and recovery. The analysis of the ATI content in five wheat species and eight cultivars each grown at three different locations in Germany revealed very low ATI amounts in einkorn. In contrast, spelt had a significantly higher total ATI content compared to modern common wheat. This was true for the two most bioactive ATIs 0.19 and CM3, as well. Because preliminary work suggests that ATI concentration and ATI bioactivity might not be correlated, further in-depth investigations using the same samples are required to establish a link between concentration and bioactivity.

## Methods

### Reagents

All reagents were of analytical grade or higher and purchased from VWR Merck (Darmstadt, Germany), Serva (Heidelberg, Germany), LECO (Kirchheim, Germany) or Sigma-Aldrich (Steinheim, Germany). Water was deionized by a water purification system Arium 611VF (Sartorius, Goettingen, Germany). Trypsin (TPCK-treated, bovine pancreas) was from Sigma-Aldrich. Peptides were synthesized by GenScript (Piscataway, NJ, USA) as unlabeled peptides (P1-P20) and as stable isotope labeled internal standards (IS1-IS20) with either two or three heavy amino acids (IS1,3,7,9,12) or *C*-terminal heavy K or R (IS2,4,5,6,8,9–11,13–20) (^13^C and ^15^N). For stock solutions (1 mg/mL in water or dimethyl sulfoxide), the peptides were solubilized according to the manufacturer’s guidelines and stored at − 80 °C prior to use.

### Grain samples

Eight cultivars representative for actual agricultural production for each of common wheat, spelt, durum wheat, emmer and einkorn were cultivated by the State Plant Breeding Institute, University of Hohenheim (Stuttgart, Germany) at three locations in Germany (Seligenstadt, Oberer Lindenhof and Eckartsweiher), and harvested in 2013 (see Supplementary Table S2). The grains were milled into wholemeal flours using a cross-beater mill (Perten Instruments, Hamburg, Germany) and stored in closed bottles for at least two weeks before analysis. The crude protein content was analyzed by the Dumas combustion method and is already reported in Geisslitz et al*.* (2019)^39^.

For method development, evaluation of precision and dilution experiments, a wheat mixture of fifteen wheat cultivars from the same location and same harvest year (2013) was used^20^. For evaluation of detection limits, extraction efficiency and dilution experiments, commercially available gluten-free wheat starch (BEZGLUTEN, Posadza, Poland) was used.

### Identification of ATI marker peptides and method development

A UniProtKB search with the keywords “amylase inhibitor” and the taxonomy *Triticeae* (taxon identifier 1648030) was performed and the amino acid sequences of twelve ATIs (Table 1) were downloaded on 28 February 2018. The amino acid sequence of the 13th ATI (WTI) was taken from Altenbach et al*.*^19^. The amino acid sequences were loaded into the targeted proteomics software Skyline (version 4.2, MacCoss Lab Software, University of Washington, Seattle, WA, U.S.A.)^40^ and the following settings were used to create in silico peptides: Digestion: Trypsin [KR|P]; 0 max missed cleavages; peptide length: Minimum 8, maximum 26; no excluded *N*-terminal amino acids; modifications: Carboamidomethyl (C), oxidation (M), Gln to pyro-Glu (*N*-term Q); max variable mods: 3; max neutral losses: 1. Transitions were predicted with the following settings: Precursor mass: Monoisotopic; product ion mass: Monoisotopic; collision energy: Thermo TSQ Vantage; declustering potential: None; optimization library: None; compensation voltage: None; optimize by transition, when present; precursor charges: 2, 3; ion charges: 1; ion types: y, b; from ion 1 to last ion-1; instrument: Min 50 *m/z*, max 1,500 *m/z*; method match tolerance: 0.6 *m/z*. A total of 15 LC–MS/MS methods in SRM mode was created with a maximum of 320 transitions per method and a scan time of 10 ms and exported by Skyline into Xcalibur (ThermoFisher Scientific, Bremen, Germany). The LC gradient was the same as reported previously^20^. Q1 and Q3 resolution was set to 0.7. A digested ATI extract of the wheat mixture, which was prepared as previously described^20^, was screened with the methods. The MS raw files were imported in Skyline and the SRM transitions of each peptide were manually analyzed to assess the signal quality and overlapping transitions at the same chromatographic retention time. Peptides with ambiguous or nonexistent peaks were excluded. At least one peptide, but ideally two peptides per ATI were selected resulting in 20 marker peptides (Table 2). The precursor (2^+^ or 3^+^) with the higher peak area was taken. The four most abundant transitions were selected based on the intensity for the peptides identified with high confidence and one product ion *m/z* higher than the precursor *m/z*. The CE was optimized for each transition by varying the CE ± 5 V. The precursor and product ions of IS1-20 were added to the method with the same SRM transitions (y and b) and CE as for P1-P20. The LC-gradient was shortened to the final gradient, the retention times for each peptide were evaluated and a timed SRM method with a scan range of ± 2 min and scan width of 10 ms was exported.

### Sample preparation for LC–MS/MS

Sample preparation was performed with small modifications as reported by Geisslitz et al*.* (2018)^20^. Flour (50 mg) was stirred twice with ammonium bicarbonate (Abic) solution (0.5 mL, 50 mmol/L, pH 7.8) for 30 min at 22 °C. After every extraction step, the suspensions were centrifuged for 25 min at 3,750 × *g* and the supernatants were combined. The extracts were evaporated to dryness in a rotational vacuum concentrator (Martin Christ Gefriertrocknungsanlagen GmbH, Osterode, Germany) and the residue was dissolved in Tris–HCl (320 µL, 0.5 mol/L, pH 8.5) and 1-propanol (320 µL). A mixture of IS1-20 (50 µL) was added for SIDA. The concentration of each IS in this solution was adjusted to the expected peptide content in the samples. Reduction was performed by adding tris(2-carboxyethyl)phosphine (TCEP, 50 µL, 0.05 mol/L TCEP in 0.5 mol/L Tris–HCl, pH 8.5) and incubating for 30 min at 60 °C. Cysteine residues were alkylated with chloroacetamide (CAA) (100 µL, 0.5 mol/L CAA in 0.5 mol/L Tris–HCl, pH 8.5) for 45 min at 37 °C in the dark. The solvent was removed by evaporation to dryness. Tryptic hydrolysis (0.5 mL, enzyme-to-substrate ratio 1:50, 0.04 mol/L urea in 0.1 mol/L Tris–HCl, pH 7.8) was performed for 18 h overnight at 37 °C in the dark. The reaction was stopped by adding 2 µL trifluoroacetic acid. The solution was diluted 1 + 1 with 0.5 mL of 0.1% formic acid (FA) and filtered through a 0.45 µm membrane.

### Response lines

Two solutions (50 µg/mL of each peptide), solution 1 with P1-P20 and solution 2 with IS1-IS20, were prepared from the stock solutions. An aliquot of each solution 1 and 2 was reduced with TCEP and alkylated with CAA as described for grain samples. Alkylated solutions 1 and 2 (10 µg/mL of each peptide) were mixed in molar ratios n(P)/n(IS) between 9.1 and 0.1 (9 + 1, 4 + 1, 3 + 1, 1 + 1, 1 + 3, 1 + 4 and 1 + 9) for calibration.

### Targeted LC–MS/MS and SIDA

An UltiMate 3000 HPLC system (Dionex, Idstein, Germany) coupled to a triple-stage quadrupole mass spectrometer (TSQ Vantage, ThermoFisher Scientific) equipped with an Aqua-C_18_ column (50 × 2 mm, 5 µm, 12.5 nm, Phenomenex, Aschaffenburg, Germany) was used for peptide separation with the following LC conditions: Solvent A: FA (0.1%, v/v) in water; solvent B: FA (0.1%, v/v) in acetonitrile; gradient: 0–18 min 5–30% B, 18–20 min 30% B, 20–21 min 30–90% B, 21–24 min 90% B, 24–25 min 90–5% B, 25–35 min 5% B; flow rate: 0.2 mL/min; injection volume: 10 μL; column temperature: 22 °C. The ion source was operated in the positive electrospray ionization (ESI^+^) mode and the following source parameters were set: Spray voltage: 4,500 V; vaporizer temperature: 50 °C; sheath gas pressure: 40 arbitrary units (au); aux gas pressure: 5 au; capillary temperature: 300 °C. Scheduled SRM was used to analyze the transitions from precursor to product ions (Table 2).

### Data analysis of SIDA

SRM peak area integration was performed using Skyline with manual verification of automated peak integration. The ratios between the five transitions were confirmed to be the same for the response lines and the flour samples (± 5% absolute deviation), allowing the use of averaged peak ratios from the five transitions, respectively. Transitions with high matrix effect were deleted with at least two remaining transitions. These constant ratios were additionally used as identification criteria for the marker peptide signals. Response lines were plotted by linear regression of the peak area ratios A(P1-P20)/A(IS1-IS20) against the molar ratios n(P1-P205)/n(IS1-IS20). All quantitations were performed from two replicates with one injection each. Contents of ATIs were calculated by multiplying the peptide content with the respective factor (M_Protein_/M_Peptide_) using the molecular mass of the protein without the signal peptide. For CM16, the protein content was calculated separately from P12/P12c and summed up. The ratio was constant at about 50/50 for P12/P12c. The content of 0.19 was obtained as difference between the sum of 0.19 and 0.53 and the content of 0.53 (P5).

### Evaluation of precision

Repeatability precision was evaluated by analysis of six replicates (n = 6) of the wheat flour mix and intermediate precision with six replicates on three days (same analyst, same instrument), respectively (n = 18)^41^.

### Evaluation of detection and quantitation limits

To determine the LOD and LOQ, P1-P20 and IS1-IS20 (500 ng, 250 ng, 100 ng, 50 ng, 25 ng, 10 ng, 5 ng, 1 ng and 0.5 ng absolute) were added to gluten-free wheat starch (50 mg) as analyte-free matrix (n = 3). Sample preparation and analysis were the same as described above. The last spike-on step, where the identification criteria (correct ratios of the five transitions) were fulfilled, was used to calculate LOD and LOQ. LOD was defined as three times the standard deviation and LOQ as ten times the standard deviation of this last step^42^. LOD and LOQ were verified by adding P1-P20 and IS1-IS20 three times each to gluten-free wheat starch at the concentrations calculated for LOD and LOQ.

### Evaluation of recovery

The recovery was defined as the recovery of the peptides’ content in the wheat flour mix compared to the sample with the wheat flour mix diluted (1 + 4) with gluten-free wheat starch.

### Statistics

One-way and two-way ANOVA with Tukey’s test (p < 0.05) and hierarchical cluster analysis were performed with Origin 2019 (OriginLab, Northampton, Massachusetts, USA). For two-way ANOVA, the factors A and B were wheat species and growing location, respectively, and interactions were enabled. Heritability was calculated as ${h}^{2}=1-\frac{\mathrm{\vartheta }}{{\sigma }_{G}^{2}}$, where ϑ is the mean variance of a difference of two best linear unbiased predictors (BLUP) and ${\sigma }_{G}^{2}$ the genetic variance^43,44^.

## Supplementary information

Supplementary Information.

Supplementary Table 2.

## Data Availability

The mass spectrometry proteomics data have been deposited to the ProteomeXchange Consortium (https://proteomecentral.proteomexchange.org) with the dataset identifier PXD020714 and are publicly available on Panorama Public (https://panoramaweb.org/2rB1TL.url). All detailed results of each analyzed protein are included in Supplementary Table S2 (online).
