# Supplementary material for: Comparative quantitative LC–MS/MS analysis of 13 amylase/trypsin inhibitors in ancient and modern Triticum species
Source: Sci Rep. 2020 Sep 3;10:14570. doi: 10.1038/s41598-020-71413-z (PMC7471314; doi:10.1038/s41598-020-71413-z)
Supplement: Supplementary file 1 — Supplementary Information. [file 41598_2020_71413_MOESM1_ESM.pdf]

Comparative quantitative LC-MS/MS analysis of 13 amylase/trypsin inhibitors in ancient and modern *Triticum* species

Supplementary Material

Sabrina Geisslitz[1,2], C. Friedrich H. Longin[3], Peter Koehler[4], Katharina Anne Scherf[1,2]

[1] Leibniz-Institute for Food Systems Biology at the Technical University of Munich, Lise-Meitner-Strasse 34, 85354 Freising, Germany

[2] Department of Bioactive and Functional Food Chemistry, Institute of Applied Biosciences, Karlsruhe Institute of Technology (KIT), Adenauerring 20 a, 76131 Karlsruhe, Germany

[3] State Plant Breeding Institute, University of Hohenheim, 70599 Stuttgart, Germany

[4] biotask AG, Schelztorstrasse 54-56, 73728 Esslingen am Neckar, Germany

Supplementary Table S1. Amino acid sequences of amylase/trypsin-inhibitors (ATIs) identified in the self-generated database with their UniProtKB accession number, their abbreviation (ATI) and the abbreviation of the marker peptide used to develop the stable isotope dilution assay (SIDA).

UniProtKB number	ATI	Amino acid sequence[1]	Peptide
P01083	0.28	SHNSGPWSW**C**NPATGYK	
P01083	0.28	VSALTG**C**R	
P01083	0.28	LQ**C**VGSQVPEAVLR	P1
P01083	0.28	D**CC**QQLADINNEW**C**R	
P01083	0.28	**C**GDLSS**M**LR	
P01083	0.28	LTAASVPEV**C**K	P2
P01083	0.28	VPIPNPSGDR	
P01085	0.19	D**CC**QQLAHISEW**C**R	
P01085	0.19	EHGAQEGQAGTGAFPR	
P01085 + P01804	0.19+0.53	LQ**C**NGSQVPEAVLR	P3
P01085 + P01804	0.19+0.53	**C**GALYS**M**LDS**M**YK	
P01085 + P01804	0.19+0.53	LTAASITAV**C**R	P4
P01085 + P01804	0.19+0.53	LPIVVDASGDGAYV**C**K	
P01085 + P01804	0.19+0.53	DVAAYPDA	
P01804	0.53	EHGVSEGQAGTGAFPS**C**R	P5
P16850	CM1	EYVAQQT**C**GISISGSAVSTEPGNTPR	
P16850	CM1	SDPNSSVLK	P6
P16850	CM1	VLVTSGH**C**NVMTVHNAPY**C**LGLDI	
P16851	CM2	EYVAQQT**C**GVGIVGSPVSTEPGNTPR	P7
P16851	CM2	TSDPNSGVLK	P8
P17314	CM3	DYVLQQT**C**GTFTPGSK	
P17314	CM3	LY**CC**QELAEISQQ**C**R	
P17314	CM3	YFIALPVPSQPVDPR	P9
P17314	CM3	SGNVGESGLIDLPG**C**PR	P10
P17314	CM3	LLVAPGQ**C**NLATIHNVR	
P16159	CM16	DYVEQQA**C**R	P11
P16159	CM16	QQ**CC**GELANIPQQ**C**R	P12
P16159	CM16	**Q**Q**CC**GELANIPQQ**C**R	P12c
P16159	CM16	EVQMDFVR	
P16159 + Q41540	CM16+CM17	SRPDQSGLMELPG**C**PR	
Q41540	CM17	NYVEEQA**C**R	P13
Q41540	CM17	IEMPGPPYLAK	
Q41540	CM17	QE**CC**EQLANIPQQ**C**R	
Q41540	CM17	**Q**E**CC**EQLANIPQQ**C**R	
P16347	WASI	AHGGGLTMAPGHGR	
P16347	WASI	**C**PLFVSQEADGQR	
P16347	WASI	IAPHGGAPSDK	
P16347	WASI	HVITGPVR	P14
P16347	WASI	YSGAEVHEYK	P15
P16347	WASI	LMA**C**GDS**C**QDLGVFR	
Q43723 + Q43691	CMX1/3+CMX2	EFIAGIVGR	P16
P83207	WCI	ELAAISSN**C**R	P17
P83207	WCI	AFPPSQSQGGGPPQPPLAPR	P18
-*	WTI	ELEAVSEE**C**R	P19
-*	WTI	**C**TAMEDFMQGMLR	
-*	WTI	LEGVPEG**C**TR	P20

[1] **C**, S-carboxamidomethylcysteine; **M**, methionine sulfoxide; **Q**, pyroglutamic acid
* Sequence according to Altenbach *et al.* (2011)[19]; similar to A0A1D5UB33 (uncharacterized wheat protein, 57 additional amino acids at the beginning and an additional T at position 173 of A0A1D5UB33)

Supplementary Table S2. Content of crude protein, content of the 13 amylase/trypsin inhibitors based on the content of the marker peptides, respectively, and total content of the sum of ATI.

Please refer to separate Excel file online

The abbreviations of peptides are according to Supplementary Table S1.

CV, average of coefficient of variation of duplicate determinations per cultivar within one wheat species.

LOD, limit of detection. For specific LODs, please refer to Table 3.

Supplementary Table S3. Correlation coefficients between the absolute content of the two marker peptides of the ATIs 0.28, 0.19, CM2, CM3, CM16, WCI and WTI for each wheat species.

**ATI**		**Common wheat**	**Spelt**	**Durum wheat**	**Emmer**	**Einkorn**
0.28	(P1/P2)	0.968***	0.997***	0.981***	0.980***	-
0.19	(P3/P4)	0.879***	0.874***	0.110	0.526*	-
CM2	(P7/P8)	0.774***	0.930***	0.954***	0.883***	-
CM3	(P9/P10)	0.899***	0.895***	0.880***	0.934***	0.948*
CM16	(P11/P12)	0.874***	0.981***	0.931***	0.059	-
WASI	(P14/P15)	0.712***	0.800***	0.768***	0.741***	-
WCI	(P17/P18)	0.985***	0.964***	-	-	-
WTI	(P19/P20)	0.999***	0.990***	0.904***	0.917***	-

Pearson correlation coefficients (r): $r \leq \pm 0.54$, no correlation; $\pm 0.54 < r \leq \pm 0.67$, weak correlation; $\pm 0.67 < r \leq \pm 0.78$, medium correlation; $\pm 0.78 < r \leq \pm 1.00$, strong correlation; level of significance (p): *, significant ($p \leq 0.05$); **, highly significant ($p \leq 0.01$); ***, very highly significant ($p \leq 0.001$).

Supplementary Table S4. *F* values of two-way ANOVA with the factors wheat species and location. Only samples with quantified levels of the respective ATI were considered and included in the test.

ATI	Absolute content			ATI distribution based on total protein content			ATI distribution based on total ATI content		
	**Wheat species**	**Location**	**Interaction**	**Wheat species**	**Location**	**Interaction**	**Wheat species**	**Location**	**Interaction**
0.28	14.2***	0.5	0.5	12.7***	4.4*	0.3	28.0***	0.9	0.1
0.19	744.4***	0.1	0.3	216.1***	4.2	4.2***	2036.7***	0.2	0.6
0.53	327.5***	17.2***	4.8***	755.0***	5.6*	2.4*	349.0***	11.7***	2.5*
CM1	15.1***	0.3	0.3	2.6	0.2	0.4	0.3	0.1	0.6
CM2	132.5***	0.0	1.6	160.9***	9.8***	2.4*	1010.5***	1.1	1.4
CM3	64.1***	0.1	1.4	59.0***	7.1**	1.9	451.7***	0.3	0.4
CM16	109.0***	0.4	0.9	104.1***	8.7***	1.6	656.9***	1.7	1.0
CM17	54.3***	0.8	0.0	9.2**	8.1**	0.2	2.7	3.7*	0.3
WASI	54.5***	6.1	1.1	58.2***	4.4*	0.6	1368.3***	2.5	2.0
CMX1/2/3	43.3***	1.7	0.5	21.5***	7.1**	0.6	1009.2***	0.9	0.4
WCI	2.9	4.5*	0.4	0.5	5.1*	0.2	7.1*	4.7*	0.2
WTI	24.1***	0.0	0.1	26.3***	0.3	0.1	23.7***	0.1	0.1
Total ATI	514.5***	0.1	1.3	426.9***	14.2***	2.2*	-	-	-

Level of significance (p): *, p ≤ 0.05, significant; **, p ≤ 0.01, highly significant; ***, p ≤ 0.001, very highly significant.

Supplementary Table S5. Comparison of amino acid sequences of ATIs reported in literature and in the UniProtKB database.

Name in literature[1]	Differences[2]	UniProtKB accession	ATI
WMAI Bu-1	38: D → N<br>145: +GD	P01083	0.28
WMAI Bu-2	2: L → W<br>12: L → V<br>38: D → N<br>139: G → R<br>145: +GD		
WDAI Bu-1	- Signal peptide	P01085	0.19
WDAI Bu-2	Different signal peptides		
WDAI Bu-3	- Signal peptide<br>40: Y → Q<br>43: K → Q<br>53: V → L<br>73: C → P<br>92: G → S<br>131: K → R<br>136: I → V<br>148: G → D	P01084	0.53
WDAI Bu-4	- Signal peptide<br>80: C → P		
WTAI-CM1 Bu-1	126: P → S	P16850	CM1
WTAI-CM2 Bu-1	/	P16851	CM2
WTAI-CM3 Bu-1	/	P17314	CM3
WTAI-CM3 Bu-2	8: N →S<br>20: V → L<br>38: D → N<br>71: F → Y<br>74: M → G<br>91: P →S<br>134: Q → E<br>165: L → P		
WTAI-CM16 Bu-1	/	P16159	CM16
WTAI-CM17 Bu-1	12: A → T	Q41540	CM17
WASI Bu-1	/	P16347	WASI
WASI Bu-2	- Signal peptide<br>91: -G<br>180: R → K		

Continued on the next page

Supplementary Table S5 Continued

Name in literature[1]	Differences[2]	UniProtKB accession	ATI
CMx Bu-1	43: A → R<br>44: C → R<br>61: E → Q<br>74: K → E<br>75: F → L<br>77: R → W<br>82: H → R<br>87: G → R<br>102: -E<br>*C*-terminus: -22 amino acids	Q43723	CMX1/3
CMx BU-3	27: D → E<br>28: R → Q<br>36: P → T<br>43: C → R<br>61: L → Q<br>64: Q → R<br>77: R → W<br>82: H → R<br>87: G → R<br>101: K → Q<br>105: G → R<br>*C*-terminus: -22 amino acids		
CMx Bu-2	2: P → A<br>8: L → I<br>13: V → I<br>17: A → V<br>21: G → A<br>27: D → E<br>28: R → Q<br>43: C → R<br>77: R → W<br>97: G → R<br>107: G → D<br>115: G → R<br>*C*-terminus: -24 amino acids	Q43691	CMX2
WTI Bu-1	*N*-terminus: +57 amino acids<br>115: +T	P81713	WTI
WCI Bu-1	- Signal peptide<br>24: M → I	P83207	WCI

[1] Altenbach, S. B., Vensel, W. H. & Dupont, F. M. The spectrum of low molecular weight α-amylase/protease inhibitor genes expressed in the US bread wheat cultivar Butte 86. *BMC Res. Notes* **4**, 242 (2011).[19]

[2] Differences of the amino acid sequence from literature → UniProtKB
